# Prevalence of cardiovascular risk factors and diseases in patients with multiple myeloma undergoing autologous peripheral blood stem cell transplantation

**DOI:** 10.18632/oncotarget.26872

**Published:** 2019-05-07

**Authors:** Deborah Backs, Ilknur Saglam, Claudia Löffler, Sandra Ihne, Caroline Morbach, Susanne Brenner, Christiane Angermann, Georg Ertl, Stefan Frantz, Stefan Störk, Stefan Knop, Gülmisal Güder

**Affiliations:** ^1^ Comprehensive Heart Failure Center Würzburg, University of Würzburg, Würzburg, Germany; ^2^ Department of Internal Medicine I, Division of Cardiology, University Hospital of Würzburg, Würzburg, Germany; ^3^ Department of Medicine II, Division of Cardiology, University Hospital of Schleswig-Holstein, Lübeck, Germany; ^4^ Department of Internal Medicine II, Division of Hematology, University Hospital of Würzburg, Würzburg, Germany

**Keywords:** cardiovascular risk factors, cardiovascular diseases, multiple myeloma, arterial hypertension, echocardiography

## Abstract

Multiple myeloma (MM) is the second most common hematologic malignancy and occurs similar to cardiovascular diseases (CVD), in the sixth/seventh decade. The aim of this retrospective cohort study was to evaluate the prevalence and prognostic value of cardiovascular risk factors (CVRF) and CVD in 325 patients with MM undergoing autologous peripheral blood stem cell transplantation (PBSCT) at the University Hospital of Würzburg between 03/2004 and 12/2011. Mean age in the total cohort was 61 years. Among CVRF, prevalence of arterial hypertension was highest (59.7%), followed by overweight (54.2%) and positive smoking history (18.2%). The prevalence of heart failure (3.1%) or coronary heart disease (4.8%) was low. During a median follow-up of 36 months, 18% of the patients died. Hypertension (HR = 1.83, *p* = 0.048) as well as positive smoking history (HR = 2.13, *p* = 0.02) were independently associated with increased mortality risk in multivariate analysis. In a subgroup analysis of 100 patients echocardiographic parameters were compared before and after PBSCT. Echocardiography revealed a significant reduction of left atrial diameters (–1.5 mm, *p* = 0.009) and septum thickness (–1.0 mm, *p* = 0.001), non-significant reduction of systolic function, and an increase of the prevalence of diastolic dysfunction (+14%; *p* = 0.01). In this study CVRF, especially hypertension and smoking, are strong predictors of poor survival in patients with MM undergoing autologous PBSCT. Echocardiography before and after treatment shows subtle changes in systolic function but an increase of the prevalence of diastolic dysfunction.

## INTRODUCTION

In Europe, cardiovascular diseases (CVD) are with 50% the most common causes of death, followed by malignant tumors in 20% of the cases [1]. General cardiovascular risk factors (CVRF) for CVD include arterial hypertension, obesity, nicotine abuse, diabetes mellitus and lipid metabolism disorders thus, optimization of those modifiable CVRF may impact on overall prognosis in the general population [2].

Associations are less clear in patients with malignant diseases. With an incidence of 86.000 cases in one year [3], multiple myeloma is currently the second most common hemato-oncological disease worldwide. Despite of a wide range of therapeutic options including high-dose chemotherapy and autologous peripheral blood stem cell transplantation (PBSCT), the disease is still incurable [4]. At the time of the initial diagnosis of multiple myeloma, patients are on average 69 years old [5]. Statistically, the prevalence of CVRF and CVD is also increasing at the 6th and 7th decade of life. At the age of > 55–65 years, arterial hypertension, the most common CVRF, has a prevalence of > 50–70% in the general population [6].

Surprisingly, the prevalence of CVRF in patients with multiple myeloma was so far reported much lower than epidemiological estimations may expect. In the cohort from which the “Freiburg Comorbidity Index” was derived [*N* = 127, median age 60 years], the overall prevalence of arterial hypertension was 16%. More importantly, occurrence of arterial hypertension or other CVRF/CVD was prognostically not relevant [7].

As multiple myeloma may not preclude CVRF, preselection bias might have confounded true associations and underestimated the relevance of CVRF and CVD [7].

Further, in recent years progress in cancer therapy has increased life expectancy despite of the use of potentially cardiotoxic substances [8, 9]. In view of the upcoming demographic changes an increase in cardiovascular morbidity and mortality may be expected that may pose greater problems, if they are not taken into account. The prediction of possible cardiovascular complications remains challenging [10].

In the current study we investigate the prevalence and prognostic value of CVRF and CVD in 325 unselected patients with multiple myeloma undergoing autologous PBSCT. In a subgroup of patients serial echocardiography was performed to assess cardiac status before and after treatment.

## RESULTS

### Patient characteristics

Taking age into account as the most relevant predictor of cardiovascular diseases, the cohort was divided in two groups (≤ versus > 60 years). All patients received autologous PBSCT at the University Hospital Würzburg between 03/2004 and 12/2011. The median follow-up was 36 months (quartiles: 21; 52 months).

Mean age in the total cohort was 61 years and 60.9% were male. Based on the classification of Salmon and Durie, 83.7% of patients were in stage II/III with signs of high disease activity (Table 1).

**Table 1:** Characterization of the cohort

	Total cohort	≤ 60 years	> 60 years	*p*-value
	*N* = 325	*N* = 142	*N* = 183	
Clinical characteristics				
**age, years**	**61 (55;67)**	**54 (47;57)**	**67 (63;69)**	**< 0.001**
masculine sex, *N* (%)	198(60.9)	83 (58.5)	115 (62.8)	0.43
Multiple myeloma stages & treatment				
Salmon & Durie stages				
1, *N* [%]	51 (15.7)	24 (16.9)	27 (14.8)	0.64
2, *N* [%]	64 (19.7)	22 (15.5)	42 (23.0)	0.12
3, *N* [%]	208 (64.0)	94 (66.2)	114 (62.3)	0.49
stage B^1^, *N* (%)	67 (20.6)	29 (20.4)	38 (20.8)	1.00
bortezomib-based induction, N (%)	17 (5.2)	9 (6.3)	8 (4.4)	0.46
**IMiD-based induction^2^, *N* (%)**	**10 (3.1)**	**8 (5.6)**	**2 (1.1)**	**0.02**
**Non novel agent-based, *N* (%)**	**298 (91.7)**	**125 (88.0)**	**173 (94.5)**	**0.04**
Clinical examination				
peripheral edema, *N* (%)	36 (11.1)	13 (9.2)	23 (12.6)	0.38
rest dyspnea, *N* (%)	3 (0.9)	1 (0.7)	2 (1.1)	1.00
Cardiovascular risk factors				
**Arterial hypertension^3^, *N* (%)**	**190 (59.7)**	**60 (43.2)**	**130 (72.6)**	**<0.001**
**defined by medical recordings, *N* (%)**	**159 (48.9)**	**50 (35.2)**	**109 (59.6)**	**<0.001**
**by antihypertensive therapy, *N* (%)**	**150 (46.2)**	**43 (30.3)**	**107 (58.5)**	**<0.001**
**blood pressure before induction > 140/90 mmHg, *N* (%)**	**101 (31.8)**	**25 (18.0)**	**76 (42.5)**	**<0.001**
BMI^4^ > 25kg/m², *N* (%)	175 (54.2)	71 (50.4)	104 (57.1)	0.26
BMI^4^ > 30kg/m², *N* (%)	48 (14.9)	24 (17.0)	24 (13.2)	0.34
positive smoking history, *N* (%)	59 (18.2)	26 (18.3)	33 (18.0)	1.00
**diabetes mellitus, *N* (%)**	**29 (9.2)**	**6 (4.3)**	**23 (13.0)**	**0.01**
hyperlipidemia, *N* (%)	52 (16.3)	20 (14.4)	32 (17.8)	0.26
positive family history^5^ *N* (%)	1 (0.3)	1 (0.7)	0 (0.0)	0.44
co-morbidities				
**heart failure, *N* (%)**	**10 (3.1)**	**0 (0.0)**	**10 (5.5)**	**0.003**
coronary artery disease, *N* (%)	15 (4.8)	4 (2.9)	11 (6.3)	0.19
peripheral artery occlusive disease, *N* (%)	3 (1.0)	0 (0.0)	3 (1.7)	0.26
pulmonary diseases^6^	18 (5.7)	4 (2.9)	14 (7.9)	0.09
renal insufficiency^7^, *N* (%)	81 (25.2)	29 (20.7)	52 (28.7)	0.12
**anemia^8^, *N* (%)**	**234 (72.0)**	**94 (66.2)**	**140 (76.5)**	**0.05**

Values are percentage of n or median (25th–75th percentile). *P*-values refer to Fisher’s exact test or the Mann–Whitney *U* test as appropriate; *p*-values ≤ 0.05 are marked (bold print).

Abbreviations: ^1^Salmon and Durie stage B = impaired renal functions with creatinine ≥ 2 mg/dl; ^2^IMiD-based induction^2^ = induction therapy with thalidomide or lenalidomide ^3^arterial hypertension = diagnosed in the doctor’s letter, in the presence of antihypertensive medication or repeatedly measured elevated blood pressure values (at least 2 of 3 measured values > 140 mmHg systolic or > 90 mmHg diastolic); ^4^BMI = Body Mass Index, ^5^positive family history = myocardial infarction before the age of 60 with parents, siblings or children; ^6^pulmonary disease = chronic obstructive pulmonary disease, bronchial asthma and restrictive pulmonary diseases; ^7^renal insufficiency = glomerular filtration rate reduction < 60 ml/min/1.73m^2^; ^8^anemia = defined as hemoglobin < 12 g/dl in women and < 13 g/dl in men.

### Cardiovascular risk factors and cardiovascular diseases

In 70.5% the patients had at least one CVRF, the prevalence was significantly higher in the group of > 60-year-olds (80.9% vs. 57.0 % compared to the group ≤ 60 years, *p <* 0.001). Among CVRF, prevalence of arterial hypertension was most common with 59.7%, followed by overweight (54.2%) and positive smoking history (18.2%). Hyperlipidemia and positive family anamnesis were less common (all Table 1).

The prevalence of cardiovascular diseases was rather low, the most common cardiac disease before treatment was coronary artery disease (4.8%), followed by heart failure (3.1%) and peripheral artery occlusive disease (1%; all Table 1).

### Medication for arterial hypertension before induction

**Figure 1:**
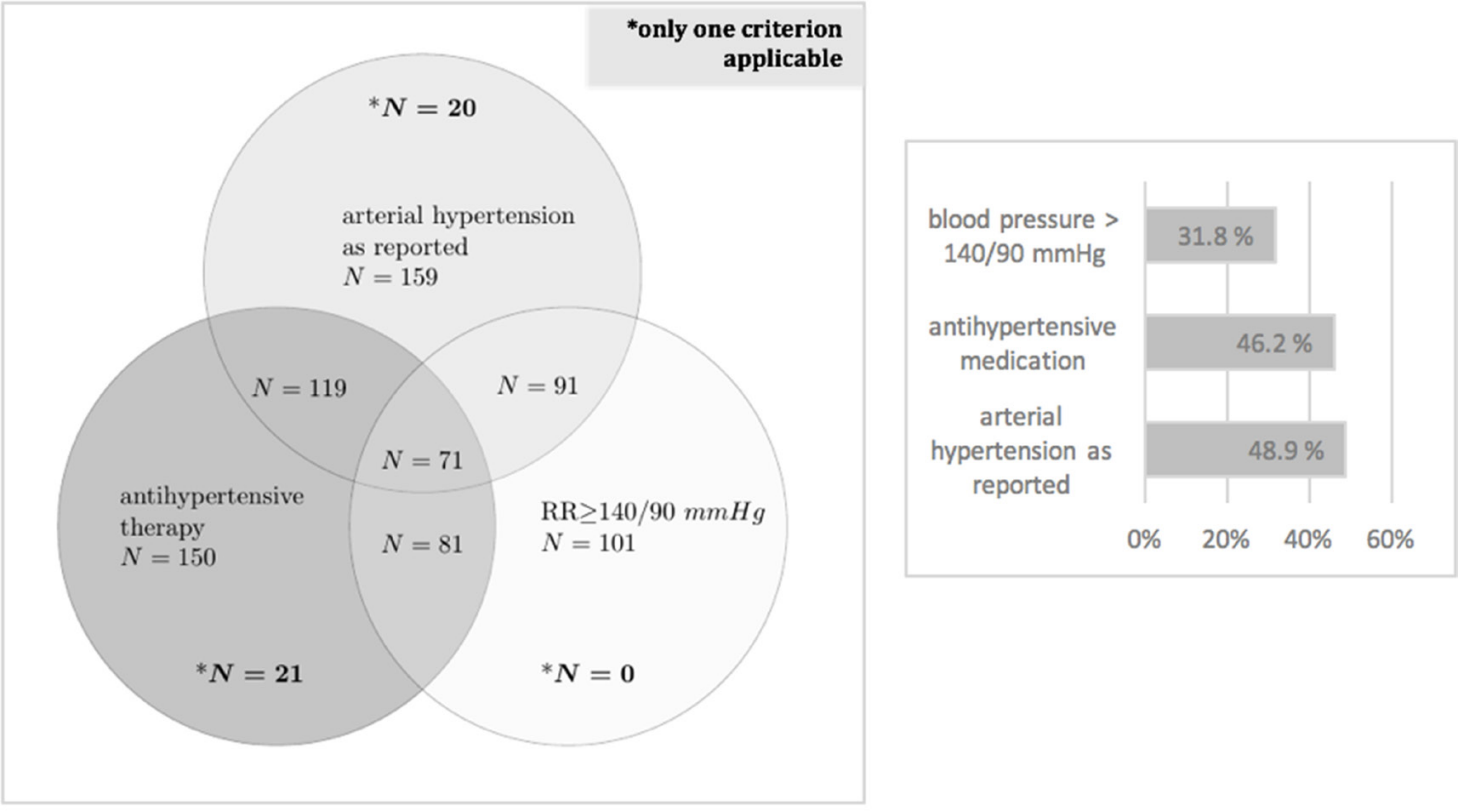
Prevalence of arterial hypertension. Diagnosis of arterial hypertension: in case of anamnestic presence of the diagnosis arterial hypertension in the doctor’s letters, administration of antihypertensive medication or hypertensive blood values prior to induction (min. 2 of 3 measured values >140 mmhg systolic/an or >90 mmHg).

In total, 150 of patients received antihypertensive treatment at admission. Less than half of them (46%; *N* = 69/150) were sufficiently controlled (blood pressure ≤ 140/90 mmHg; Figure 1). Further only 75% (*N* = 119/159) of patients with reported arterial hypertension received therapy. Angiotensin-converting-inhibitors and angiotensin-antagonists were the most commonly used compounds with 27.1%, followed by beta-blockers (21.8%) and calcium-antagonists (15.4%). Mineralocorticoid receptor blockers were found in only 2.1% of patients.

### Medication for other cardiovascular diseases before induction

Antidiabetic therapy was prescribed in 62.1% of patients with diabetes (*N* = 18/29). Hyperlipidemia was present in 16.3% of the cases and 63.5% (*N* = 33/52) of the patients received a guideline-oriented therapy with a statin.

### Other concomitant disease

Renal insufficiency, defined according to KDOQI (Kidney Disease Outcomes Quality Initiative), was present in 25.2% of patients before induction therapy (median estimate GFR 89 ml/min/1.73 m). In 6.9% of the cases a stage 3 (defined as an estimate GFR < 30 ml/min/1.73 m) was present before induction (Table 1).

Prior induction, 72% were diagnosed with anemia (defined as hemoglobin (Hb) < 12 g/dl in women, < 13 g/dl in men). In the group of patients > 60 years median Hb of was 11.2 g/dl (Table 1).

Pulmonary diseases (such as chronic obstructive pulmonary disease, COPD, bronchial asthma and restrictive pulmonary diseases) were reported in 5.7% and were more frequently in the group of patients over 60 years (Table 1).

### Analysis of survival

During a median follow-up time of 36 months (quartiles 21; 52), 18% (*N* = 59/325) of the patients died. In 50.2% (*N* = 30/59) of the cases, the cause of death was septic multiorgan failure; half of them caused by pneumonia (*N* = 16/30; 53.3%). In total, 8 patients (14%) died of a cardiovascular cause: 5.1% of chronic heart failure (*N* = 3/59), 3.4% suspect of cardiac amyloidosis (*N* = 2/59), 3.4% of malignant heart rhythm disturbances (*N* = 2/59) and 1.7% of stroke (*N* = 1/59); all of them suffered from arterial hypertension.

### COX regression analysis

All variables listed in Table 1 were analyzed. In univariate Cox regression analysis dyspnea at rest, arterial hypertension, positive smoking history, peripheral artery occlusive disease, renal insufficiency and anemia were significant predictors of death (Table 2).

**Table 2:** Univariate Cox regression analysis of patient’s characteristics

	Hazard ratio	CI 95%	*p*-value
Clinical characteristics			
age, per year	1.00	0.97–1.03	0.79
masculine sex, yes vs no	1.25	0.74–2.10	0.41
Multiple myeloma stages and treatment			
Salmon & Durie stages			
1	1.00	0.50–2.03	1.00
2	0.62	0.30–1.26	0.19
3	1.42	0.82–2.49	0.20
**stage B, yes vs no**	**2.37**	**1.38–4.07**	**0.002**
bortezomib-based induction, yes vs no	0.99	0.31–3.20	0.99
IMiD-based induction, yes vs no	0.39	0.53–2.81	0.27
Non novel agent-based, yes vs no	1.42	0.51–3.92	0.50
Clinical examination			
peripheral edema, yes vs no	0.90	0.32–2.51	0.84
**rest dyspnea, yes vs no**	**8.91**	**2.12–37.4**	**0.003**
Cardiovascular risk factors			
Body Mass Index> 30kg/m, yes vs no	1.19	0.51–2.78	0.69
**Arterial hypertension^1^**, **yes vs no**	**1.93**	**1.11–3.36**	**0.02**
**diagnosis before induction**	**1.76**	**1.05–2.94**	**0.03**
**antihypertensive therapy**	**1.76**	**1.05–2.98**	**0.03**
blood pressure > 140/90 mmHg before induction	1.25	0.73–2.14	0.43
**smoking history, yes vs no**	**1.98**	**1.10–3.58**	**0.02**
diabetes mellitus, yes vs no	0.88	0.32–2.44	0.80
hyperlipidemia, yes vs no	1.15	0.54–2.45	0.72
positive family history, yes vs no	7.00	0.96–51.2	0.06
co-morbidities			
heart failure, yes vs no	1.83	0.44–7.58	0.40
coronary artery disease, yes vs no	1.05	0.26–4.34	0.94
**peripheral artery occlusive disease, yes vs no**	**12.63**	**2.92–54.5**	**0.001**
pulmonary diseases, yes vs no	2.35	0.84–6.56	0.10
**renal insufficiency, yes vs no**	**2.03**	**1.19–3.47**	**0.01**
**anemia**, yes vs no	**2.06**	**1.07–3.96**	**0.03**

All variables of Table 1 were tested; associations with *p* ≤ 0.05 are marked (bold print). Abbreviations and definitions as in Table 1.

**Figure 2:**
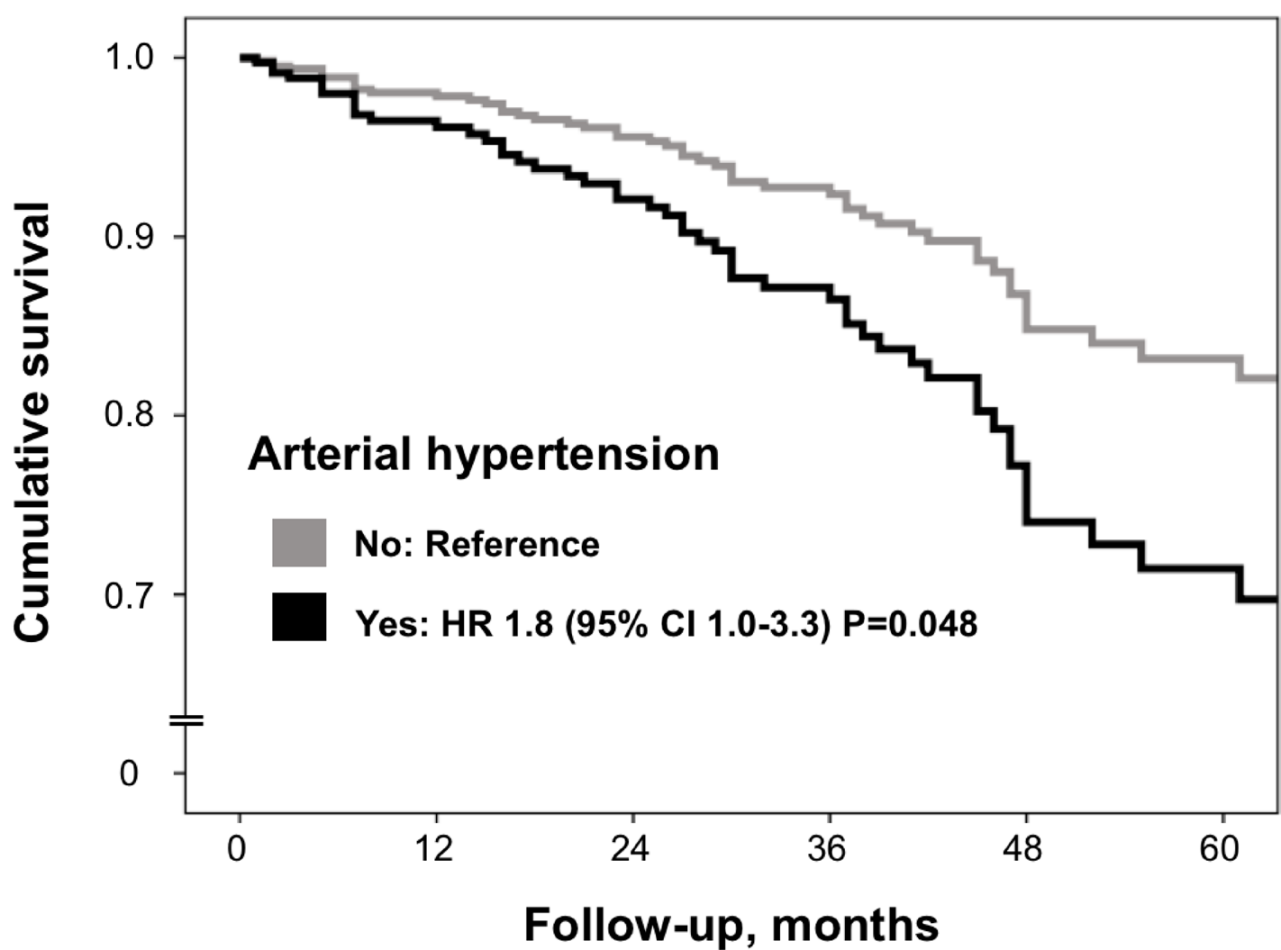
Multivariate Cox regression analysis. Definitions as in Table 1. All significant predictors (p<0.05) from univariate Cox regression (Table 2) were included. Age and sex were forced into the model. To avoid multi-collinearity, the variable "Salmon and Durie stage B" (creatinine ≥ 2 mg/dl) but not renal insufficiency (glomerular filtration rate reduction < 60 ml/min/1.73m2) was included into the model.

Arterial hypertension (HR = 1.83, *p* = 0.048; Figure 2) as well as smoking history (HR = 2.13, *p* = 0.02) were independently associated with increased mortality risk in multivariate Cox regression analysis adjusted for significant (*p* ≤ 0.10) predictors of death in univariate analysis (Table 2 age and sex forced into the analysis).

Other independent predictors of death in the multivariate Cox regression analysis were renal insufficiency (HR = 2.28, *p* = 0.004) and peripheral arterial occlusive disease (HR = 10.4, *p* = 0.03; Table 3).

**Table 3:** Multivariate Cox regression analysis

	Hazard Ratio	CI 95%	*p*-value
age, per year	0.99	0.95–1.02	0.37
masculine sex, yes vs no	1.46	0.84–2.53	0.18
**arterial hypertension^1^, yes vs no**	**1.83**	**1.01**–**3.31**	**0.048**
**smoking history, yes vs no**	**2.13**	**1.12**–**4.05**	**0.02**
**peripheral occlusive arterial disease, yes vs no**	**10.4**	**1.23**–**89.1**	**0.03**
anemia, yes vs no	1.98	0.99–3.93	0.052
**Salmon and Durie stage B^2^, yes vs no**	**2.28**	**1.30**–**4.00**	**0.004**
dyspnea at rest, yes vs no	1.86	0.23–15.10	0.56

Definitions as in Table 1. All significant predictors (*p* < 0.05) from univariate Cox regression (Table 2) were included. Age and sex were forced into the model. To avoid multi-collinearity, the variable “Salmon and Durie stage B” (creatinine ≥ 2 mg/dl) but not renal insufficiency (glomerular filtration rate reduction < 60 ml/min/1.73 m^2^) was included into the model.

### Subgroup-analysis of patients with serial echocardiography

In a subgroup of 100 patients, serial echocardiography was available before and after induction therapy (Table 4). During a median follow-up time of 13 months (quartiles: 7; 27 months) a significant reduction of left atrial diameters (LADs: –1.5 mm, *p* = 0.009) and septum thickness (–1.0 mm, *p* = 0.001) was observed. There were no changes concerning left ventricular ejection fraction (LVEF; before/after therapy LVEF ≥ 55%, *p* = 0.24) but the prevalence of patients with diastolic dysfunction increased by 16.5% (69.4% before and 85.9% after treatment, *p* = 0.01). The E/A-ratio showed a significant decrease (*p* = 0.04). There was no evidence of higher-grade valvular heart disease.

**Table 4:** Serial echocardiography before and after induction

	*N*	before induction	*N*	after induction^1^	*p*-value
**LADs (mm)**	**82**	**38.0 (34.8; 40.0)**	**82**	**36.5 (32.0; 40.0)**	**0.009**
LVDs (mm)	50	28.0 (24.8; 32.0)	50	28.0 (25.0; 33.0)	0.41
LVDd (mm)	86	47.0 (42.5; 52.0)	86	46.0 (42.0; 51,0)	0.62
LVPWd (mm)	72	10,0 (9.0; 11.0)	72	10.0 (9.0; 11.0)	0.49
**septum thickness (mm)**	**85**	**10.0 (9.0; 12.0)**	**85**	**9.00 (9.0; 11.0)**	**0.001**
posterior wall (mm)	64	10.0 (9.0; 11.0)	64	10.0 (9.0; 11.0)	0.41
LVEF (%)	100	55 (55; 64)	100	55 (55; 60)	0.24
**E/A-ratio**	**71**	**0.9 (0.8; 1.2)**	**71**	**0.8 (0.7; 1.0)**	**0.04**
deceleration time (msec)	74	200 (171; 248)	74	213 (183; 269)	0.14
**diastolic dysfunction *N* (%)**	**85**	**59 (69.4)**	**85**	**73 (85.9)**	**0.002**

^1^Measurements were performed at least ≥ 2 months after starting induction therapy; (median, 25th; 75th percentile; *N* = number of patients; *p* value ≤ 0.05 as significant). Values are median (25th–75th percentile) or percentage of N; *p*-values refer to Mann–Whitney *U* test or Fisher’s exact test as appropriate.

Abbreviations: LADs: left atrial diameter systolic; LVDs: left ventricular diameter systolic; LVDd: left ventricular diameter diastolic; LVPWd: left ventricular posterior wall end diastolic diameter; LVEF: left ventricular ejection fraction.

### COX regression of the subgroup analysis

In the subgroup of *N* = 100 patients with available echocardiography 14 patients died, thus multivariate analysis was not reliable in this subgroup.

In univariate Cox regression analysis, a significant survival benefit could only be demonstrated for LVEF (HR = 0.89, 95% CI (0.80–1.00) *p* = 0.05) but not for other echocardiographic parameters as diastolic function or cardiac diameters (data not shown).

## DISCUSSION

In this retrospective study on 325 patients with symptomatic multiple myeloma undergoing autologous stem cell transplantation we could show that in the majority of patients at least one cardiovascular risk factor was present prior to the beginning of the treatment (71.1%, *N* = 231/325) in most cases arterial hypertension (*N* = 190, 59.7%). In addition, we could demonstrate, that the cardiovascular risk factors arterial hypertension (HR: 1.83, *p* = 0.048) and positive smoking history (HR: 2.13, *p* = 0.02) were associated with a nearly twofold higher risk of death in multivariate Cox regression analysis. Serial echocardiography showed, that diastolic but not systolic function worsened significantly over time, despite of a decrease of left atrial diameters and left ventricular septum thickness.

### Risk scores in multiple myeloma and cardiovascular risk factors and diseases

Multiple myeloma most frequently occurs between the sixth and seventh decade [5], an age period generally associated with a high comorbidity burden [11].

Higher comorbidity burden predicts worse outcome and worse therapy tolerance [11]. To guide personalized treatment strategies, risk models as the “Charlson Comorbidity Index” (CCI) [12] or the “Freiburg Comorbidity Index” (FCI) [7], were developed from forecast-relevant co-morbidities in multiple myeloma [13].

The 19 co-morbidities of the CCI include diabetes mellitus, cardiac insufficiency, and myocardial infarction as cardiovascular diseases; but not for instance hypertension or smoking history [12, 13]. The FCI considers only 3 determinants: physical capacity, renal insufficiency and lung diseases [7, 14]. Other comorbidities like cardiac diseases and hypertension were tested but showed no relevant mortality-association, neither in uni- nor in multivariate Cox regression analysis [7]. The FCI was revised later, and age and frailty were included into the model (“revised Multiple Myeloma index”) [15]. The prognostic value of cardiac diseases but not of hypertension was reevaluated in this study and again no association was seen [15].

In our study, heart failure and coronary artery disease were also not predictive.

*Kim et al.* compared the CCI and the FCI risk scores in a retrospective study of 127 patients aged 65 years and older with a primary diagnosis of multiple myeloma [13]. The prevalence of CVD was—equal to the *AmcorRetro* study—rather low (coronary heart disease/myocardial infarction: 5.5% and chronic heart failure: 5.5%) [13].

The lack of prognostic impact of cardiac diseases might be also explained by the low numbers of affected individuals in our and the described cohorts [7, 13]. In a larger cohort of patients with multiple myeloma (*N* = 2190), co-incidence of heart failure (prevalence 5.9%) and a history for myocardial infarction (prevalence 5.4%) were both associated with an increased mortality risk [11].

Of note, *Engelhardt et al*., who studied a cohort of 801 patients with multiple myeloma, did not see a mortality association but described a prevalence of 45% for cardiac diseases [15]. However, cardiac diseases (defined as arrhythmias, myocardial infarction/CAD, heart failure) were subsumed for analysis, thus direct comparison may not be feasible [15].

Differences in the definition of hypertension might also explain the discrepancies concerning the prevalence of hypertension and its predictive value in patients with multiple myeloma. In the study of *Kleber et al*., the prevalence of hypertension was 16% and thus more than three times lower than in our study (59.7%) despite of a similar age distribution (median age 60 [7] vs. 61 years in our study). A definition for hypertension was not given [7].

According to a register by *Piccirillo et al.,* arterial hypertension is the most common co-morbidity in patients with malignancies (hypertension prevalence 38%) [16].

The German “CARLA-Study” (Cardiovascular Disease, Living and Ageing in Halle), a prospective cohort study that included community-dwelling adults, observed a hypertension prevalence of 50–70% in the age group of 55–65 years [6]. The German *MONICA*-*Study*, reported a similar prevalence of 60–70% in the age group of 65–74 years and a prevalence of 55–60% in the age group of 55–64 years in unselected subjects from the general population [17].

Considering that 56% of the patients in the present study were older than 60 years and that the incidence of arterial hypertension increases with age, the prevalence of arterial hypertension in our study corresponds exactly to the prevalence in the general population in Germany [18].

Of note, our data were derived from patients receiving PBSCT from 2004–2011. As life expectancy for patients with multiple myeloma increased under modern chemotherapy, and as age is one of the most important determinants of CVRF and cardiovascular diseases, the implications of accurate cardiovascular risk control may have become even more important in recent years [8].

Our results suggest that hypertension is a so far underestimated risk factor in patients with multiple myeloma, that needs to be acknowledged more accurately not only in clinical practice but also in future risk prediction models.

### Cardiovascular risk factors and death

High blood pressure was recently identified as a risk factor for chemotherapy-induced cardiotoxicity [19]. In our study coincidence of arterial hypertension doubled mortality risk in patients with multiple myeloma.

Overall, 79.3% of patients with a previously reported diagnosis of arterial hypertension were treated with anti-hypertensive therapy (119/159). But less than half of them (71/159) were sufficiently controlled (Figure 1). Early antihypertensive medication and regular cardiological check-ups are recommended in patients after stem cell transplantation [20] but should also be acknowledged before therapy initiation [9].

In our study 18.2% of all patients had a positive smoking history. A positive smoking history doubled mortality risk in multivariate Cox regression analysis. The most common cause of death in these patients was in the context of pneumonia or pneumonic sepsis, which is consistent with findings from previous studies [21]. Tobacco use is not only a cardiovascular but also a pulmonary risk factor and predictor of underlying lung disease [22–24]. Lung diseases and abnormalities in pulmonary function testing are among the most important determinants of death in patients with multiple myeloma [7, 15, 25]. Thus patients with a positive smoking history are at higher risk for both, adverse pulmonary as well as cardiovascular events.

*Tran et al.* demonstrated a correlation between the respiratory failure after (allogeneic) stem cell transplantation within 100 days of transplant and the number of smoked cigarettes (“packyears”) [26]. The authors of the study called for further studies to track the late effects of tobacco use under therapy. They were also in favor of including smoking status before and after transplantation as a relevant predictive factor into general risk assessment scores.

In view of the general data and study results, it is assumed that smoking cessation may improve survival in patients with hemato-oncological diseases. The chemotherapy induced lung toxicity seen also in multiple myeloma [27], can add up to the risks of smoking, so that nicotine withdrawal is already recommended [28] and pulmonary function monitoring by some authors [27].

### Echocardiography

Serial echocardiography before and after autologous PBSCT revealed a significant reduction of left atrial diameters (LADs: –1.5 mm, *p* = 0.009) and septum thickness (–1.0 mm, *p* = 0.001). Higher values of LADs and septal thickness are two of many indicators of worse diastolic function and routinely seen in cardiac amyloidosis as well as in hypertension [29, 30]. While the observed reduction of LADs might be simply explained by an intended volume overload before chemotherapy-initiation, reduction of left ventricular septal diameters might be an actual therapy effect and reflect reduction of para-protein deposition or amelioration of blood pressure control. However, both explanatory approaches have to be seen as highly speculative and based on the rather small number of patients in the subgroup (*N* = 100) simple chance finding cannot be excluded.

*Raina et al.* demonstrated in his study, that LVEF of patients with multiple myeloma and cardiac amyloidosis could be improved after stem cell transplantation [31].

We found no changes in mean or median left ventricular ejection fraction (LVEF before/after therapy ≥ 55%, *p* = 0.24) but 3 patients newly developed left ventricular dysfunction defined as a LVEF of < 50% after therapy (data not shown). Further, LVEF was the only echocardiographic parameter found, with a significant predictive value in Cox regression. The prevalence of patients with diastolic dysfunction increased by 16.5% (*p* = 0.02) in our study. *Karvandi et al.*, also demonstrated in 30 patients with different hemato-oncological diseases that induction therapy with subsequent stem cell transplantation can have a negative influence on diastolic function [32]. However, in our study, presence of diastolic dysfunction was not predictive and therapy modalities for multiple myeloma have changed in the last decade. Thus clarification of the true prevalence and prognostic significance of a deteriorating diastolic function after chemotherapy is reserved for prospective studies with larger cohort sizes.

### Limitation

The limitation of our study resulted from the retrospective and thus partly non-standardized data collection. Further, missing values can lead to information gaps and thus to distortions of the results. The cohort examined included consecutive patients of a university center; thus already selected patients. Since there are only a few myeloma centers in Germany, and Würzburg is one of the larger centers, it can be assumed that the results can be transferred to the group of myeloma patients eligible for autologous PBSCT in general.

As data collection was between 2004 and 2011, and standard therapy for multiple myeloma changed thereafter, results from echocardiography analysis may not be generalizable to current multiple myeloma cohorts. Further, subgroup analysis was limited by a small number of documented follow-up echocardiographs. This was due to the lack of electronic echocardiography documentation in this period due to a system change. However, age, sex and mortality rate were rather similar between the total cohort and the subgroup (data not shown), thus results of echocardiography analysis still might be representative.

## MATERIALS AND METHODS

The *AmcorRetro* study is a retrospective observational study evaluating the prevalence of CVRF and other CVD with multiple myeloma necessitating autologous PBSCT.

In total 325 patients (age range 33 to 80 years) with the diagnosis of multiple myeloma, who had at least one autologous PBSCT in Würzburg during the years 2004 to 2011 were included. Multiple myeloma was defined according to the recommendations of *the International Myeloma Working Group* [33]. The patients were identified using the Adult Stem Cell Transplantation Program of the University Hospital of Würzburg. The median follow-up was 36 months (quartiles 21; 52). All patients were followed by the outpatients department of our oncology center. If a patient died, a note including the most probable death cause was documented in the recordings. To evaluate the influence of CVRF and CVD, there was a subdivision into cardiovascular and non-cardiovascular causes of death. Survival status of the patients was assessed until 31 of December 2013.

Data were acquired retrospectively using the digitized patient files. Approval of the ethics committee was seeked and deemed as unnecessary due to the retrospective nature of the study design.

### Data acquisition and case report forms

Based on the digitized patient records, the following patient characteristics were collected at the beginning of the initiation of induction therapy using standardized case report forms: anamnesis with clinical findings (with emphasis on signs of heart failure), CVRF and CVD including other concomitant diseases (Table 1), medication (with emphasis on the therapy of CVD and CVRF), further examinations (e.g. imaging diagnostics, laboratory parameters, ECG) and the subgroup analysis of echocardiography.

Arterial hypertension was diagnosed if arterial hypertension was reported in the doctor’s letter, in the presence of antihypertensive medication (angiotensin-converting-enzyme-inhibitors, angiotensin-receptor-antagonists, beta-blockers, mineralocorticoid receptor blocker and calcium-antagonists) or repeatedly measured elevated blood pressure values (at least 2 of 3 measured values > 140 mmHg systolic or > 90 mmHg diastolic) the day after hospital admission.

### Statistical evaluation

The statistical evaluation was performed using *SPSS Statistics* (version 23). The descriptive statistics included the use of position (mean, median, minimum, maximum) and scattering measures (standard deviation, 25th/75th percentile). Group comparisons were carried out for nominal and ordinal parameters using exact Fisher or Chi-square tests (for > 2 groups) and for metric parameters using Mann–Whitney *U*-tests. To identify prognostic determinants, a univariate Cox regression analysis was carried out. With the results of the univariate analysis (with *p*-value < 0.10) a multivariate analysis was carried out taking into account statistically significant parameters. A significant group difference was assumed for all test procedures at a (two-sided) *p*-value of < 0.05.

### Subgroup analysis serial echocardiographic measurements

For the subgroup analysis all patients with at least two serial echocardiographic assessments (one before, one after induction therapy) were included (*N* = 100 patients).

Echocardiography was performed according to guidelines [29] and focused on the following measurements: left ventricular systolic function (LVEF), left atrial diameter measured at the end-systole (LADs), left ventricular end-diastolic diameter (LVDd), septal and posterior wall diameter, diastolic dysfunction (ratio E/A, E/E’, deceleration time), the presence of (indirect) signs of cardiac amyloidosis (pericardial effusion, granular sparkling) and valvular diseases. The LVEF was assessed either by the biplane-modified Simpson method or visually by eye balling in the apical four-chamber view in cases of poor ultrasound conditions.

LV end-diastolic diameter (LVEDD), LVEF, valvular function and systolic tricuspid valve gradient were assessed and graded according to the practice guidelines [29, 34].

Since not all diastolic functional parameters were included in the echocardiographic report as standard at the time of data collection, diastolic dysfunction was diagnosed if it was mentioned in the written echocardiography report (on the basis of the quantitative information provided by the investigator).

### Missing values

Due to the retrospective study design, the documentation of values was in some cases incomplete. Body weight was not documented in one patient, blood pressure measurements and smoking status were missing in 7, and oral antidiabetics in 11 patients. The numbers of missing echocardiographic parameters were shown in Table 4.

## CONCLUSIONS

In summary, with a total of 325 patients, the AmcorRetro study is currently one of the largest studies investigating the prevalence and prognostic significance of cardiovascular risk factors and diseases of patients with multiple myeloma. The study demonstrated, that especially arterial hypertension and smoking are strong predictors of poor survival in patients with symptomatic multiple myeloma undergoing peripheral autologous stem cell transplantation. Serial echocardiography before and after treatment shows that autologous PBSCT impairs systolic function only slightly, but increases the prevalence of diastolic dysfunction—underlining the need for a strict cardiological monitoring during the course of therapy in multiple myeloma.

## Abbreviations

CCI: Charlson Comorbidity Index
CI 95%: 95% confidence interval
CVD: cardiovascular diseases
CVRF: cardiovascular risk factors
FCI: Freiburg Comorbidity Index
Hb: hemoglobin
LADs: left atrial diameter systolic
LVDd: left ventricular diameter diastolic
LVDs: left ventricular diameter systolic
LVEF: left ventricular ejection fraction
LVPWd: left ventricular posterior wall end-diastolic diameter
PBSCT: peripheral blood stem cell transplantation
